# Dyspepsia: Its Varieties and Treatment

**Published:** 1910-11

**Authors:** 


**Dyspepsia: Its Varieties and Treatment.** By **W. Soltau Fenwick,** M.D. (London), Doctor of Medicine of the University of Strassburg. Octavo of 485 pages. Illustrated. Philadelphia and London. W. B. Saunders Company. 1910. (Cloth, $3.00 net.)

The writer states that the present volume is the outcome of the clinical experience gained by the personal examination of eighteen thousand cases suffering from indigestion. The claim is made that out of 100 cases of chronic dyspepsia in persons over 65 years of age, sixty-six of the cases are secondary to organic disease of some other viscera. The author warns those physicians who would succeed in the treatment of indigestion that " The stomach is not a stewpan or a test tube, but a stomach." The causes, pathology, symptoms, diagnosis, prognosis and treatment of the various forms of dyspepsia are discussed with clearness and directness. The work treats of a most important subject and is of great practical value. J. A. R.

